# Bilateral Avascular Necrosis of the Femoral Heads Secondary to Familial Hyperlipidemia

**DOI:** 10.7759/cureus.44910

**Published:** 2023-09-08

**Authors:** Thomas F Fusillo, Michael Nguyen

**Affiliations:** 1 Internal Medicine, Icahn School of Medicine at Mount Sinai, New York, USA; 2 Medicine, College of Osteopathic Medicine, Des Moines University, Des Moines, USA; 3 Sports Medicine, Capital Orthopaedics & Sports Medicine, Des Moines, USA

**Keywords:** walk-in clinic, pelvic anatomy, orthopedic sports medicine, statin use, hyperlipidemia, osteonecrosis of the femoral head, avascular necrosis (avn)

## Abstract

Avascular necrosis (AVN) is a progressive disease characterized by bone death secondary to an interruption of the relevant vascular supply. While it is most common in pediatrics and later adulthood, it can occur at any age. This case describes a previously healthy man in his mid-twenties who presented with worsening hip pain. Imaging, including X-ray and magnetic resonance, revealed severe marrow edema and early collapse of the femoral head. The patient was also found to have a severely elevated low-density lipoprotein level, leading to the diagnosis of AVN due to familial hyperlipidemia. He received a total hip arthroplasty and was started on high-intensity statin therapy. This case highlights the importance of considering AVN in the young adult population with hip pain as well as the appropriate workup and treatment.

## Introduction

Avascular necrosis (AVN) of the femoral head represents the majority of AVN cases, with other common locations including the humerus, knee, talus, lunate, scaphoid, and jaw [1]. AVN is primarily caused by either genetic predisposition or various lifestyle elements such as alcohol abuse, corticosteroid use, and trauma [2]. This has led to it having a somewhat bimodal age distribution with higher instances of occurrence in pediatric populations and adults over the age of 40 [3,4]. More common pathologies responsible for hip pain in young adults include musculotendinous strain, ligamentous sprain, contusion, and bursitis. Thus, these should be ruled out initially [5]. However, AVN should remain a consideration when working up hip pain, especially anterior hip pain, regardless of patient demographics. There is also always a chance of there being a previously undiagnosed hereditary or genetic predisposition, such as in the case of our patient. Finally, as seen with our patient, AVN can be a bilateral and asymptomatic process.

## Case presentation

A man in his mid-twenties with no significant medical history presented to the sports medicine walk-in clinic with worsening left hip pain. The pain had been progressing over the past three months, was located in the inguinal crease, did not radiate anywhere, was stabbing in nature, provoked by putting any weight on the joint (walking, running, etc.), and was mildly palliated by rest from activity. The patient had tried using ibuprofen, heat, and ice, none of which provided any substantial relief. He didn’t take any medications and didn’t have any surgical history. He was a graduate student, no smoker, had 3-5 servings of alcohol a week, and endorsed a standard American diet. He had a normal BMI, denied any trauma to the area, and was not overly active. Additionally, he denied any prior use of corticosteroids, scuba diving, alcohol abuse, and family history of any clotting disorders. However, there was a family history of hyperlipidemia and coronary artery disease.

On exam, the passive and active range of motion of the left hip joint was severely limited due to pain, especially with hip flexion and external rotation. Left hip strength was also moderately reduced secondary to pain. Straight leg raise of 20 degrees. Gait assessment revealed a moderate left-sided limp. Physical exam of the right hip was grossly normal.

Investigations

**Figure 1:**
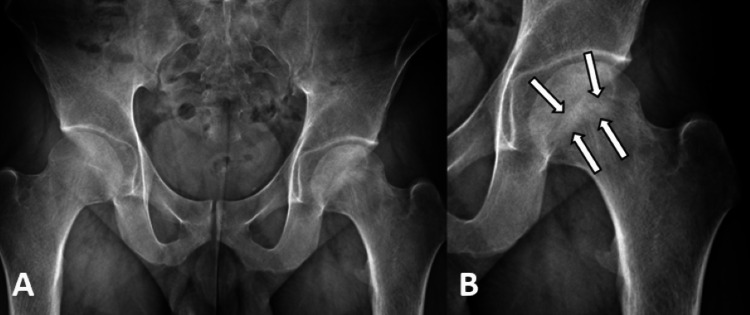
AP plain film of the pelvis (A) and left hip joint (B) Arrows pointing toward a sclerotic band on the left femoral head. AP: Anteroposterior

**Figure 2:**
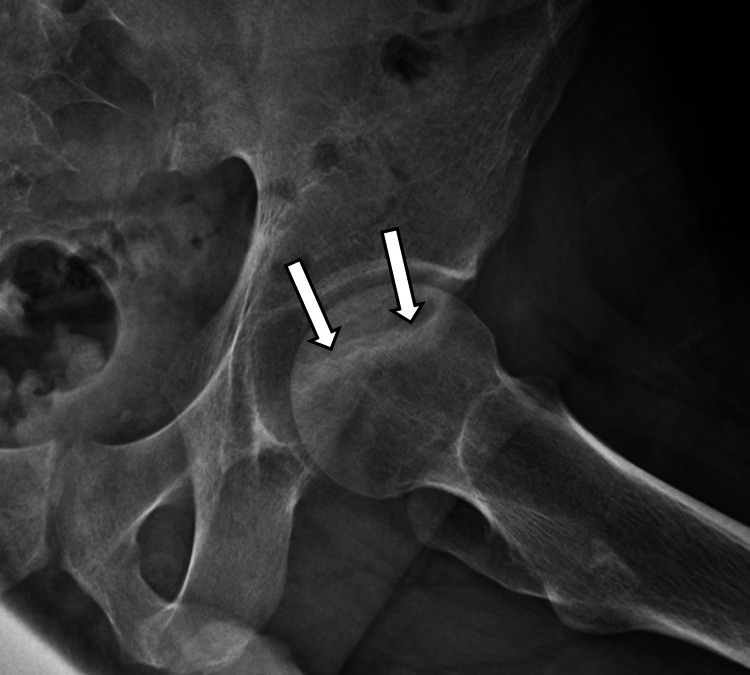
AP plain radiograph frog-leg view of the left hip Arrows pointing toward a sclerotic band on the left femoral head. AP: Anteroposterior

**Figure 3:**
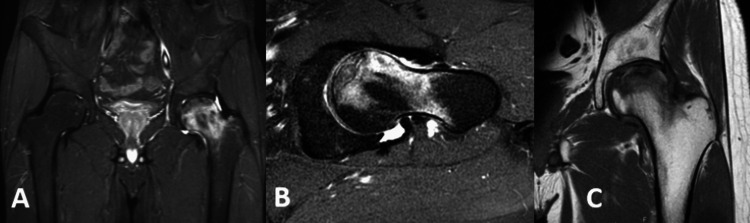
MRI pelvis A: FLAIR pelvis coronal view showing severe marrow edema of the left femoral head. B: Left hip axial view showing diffuse marrow edema, early subchondral collapse, and resultant joint effusion. C: Left hip T1 coronal view showing diffuse marrow edema with early subchondral collapse. FLAIR: Fluid attenuated inversion recovery

During the initial encounter, anteroposterior (AP) pelvis, left hip, and frog leg X-rays were taken (see Figure 1). These revealed an abnormal sclerotic band on the anterior surface of the left femoral head, best visualized on the frog leg view (see Figure 2). Magnetic resonance imaging (MRI) of the pelvis without contrast was then ordered which revealed an infarct of the left femoral head with very early subchondral collapse as well as extensive adjacent bone marrow edema (see Figure 3). These findings were consistent with AVN. The MRI also revealed a tiny infarct in the right femoral head, suggesting a bilateral process.

Further workup to identify the cause included a complete blood count, complete metabolic panel, hemoglobin A1c, c-reactive peptide, erythrocyte sedimentation rate, prothrombin time, and partial thromboplastin time, all of which were unremarkable. Additionally, a lipid panel revealed a total cholesterol of 279 mg/dL, high-density lipoprotein (HDL) of 47 mg/dL, low-density lipoprotein (LDL) of 190 md/dL, and triglycerides of 210 mg/dL.

Differential diagnosis

Prior to obtaining any imaging, the differential diagnosis included iliopsoas bursitis and femoroacetabular impingement (FAI), in addition to AVN. The iliopsoas bursa lies between the musculotendinous junction of the iliopsoas and the hip joint capsule [6]. In the normal state, it is collapsed but can become inflamed after trauma or periods of overuse. Pain from iliopsoas bursitis often radiates toward the ipsilateral knee or back and can be elicited by palpation over the bursa [6]. However, our patient denied any radiation of the pain and we could not illicit any pain on palpation, making this diagnosis less likely.

FAI is a condition in which a portion of either the acetabulum or femoral head develops spurs that irritate the normal ball-in-socket motion of the hip joint. Traditionally, this causes pain with internal rotation of the hip and is exacerbated by sitting with the hip in flexion [7]. Our patient had more pain with external rotation and actually found relief from sitting with the hip in flexion. Additionally, there was no spurring or bone overgrowth seen on the initial X-rays.

Treatment

The patient received a left total hip arthroplasty (THA) followed by an immediate two weeks of physical therapy. He was also started on high-intensity statin therapy with atorvastatin 40mg once daily.

Outcome and follow-up

**Figure 4:**
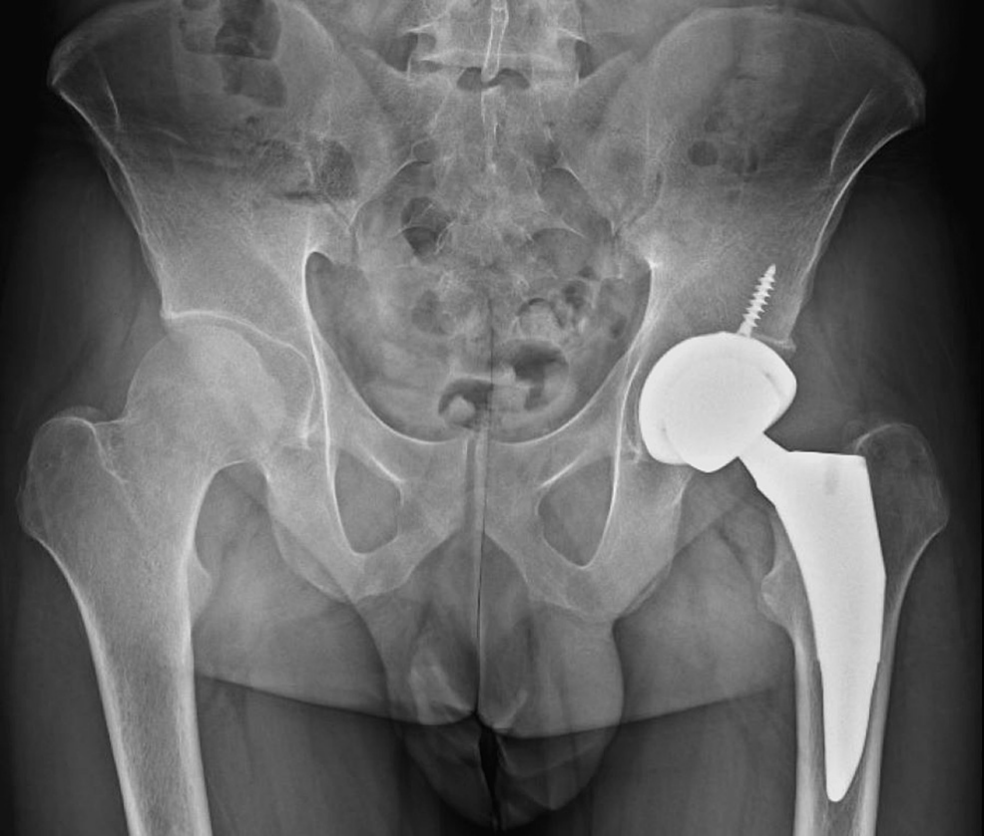
AP plain film one month post-operative revealing prosthesis in good position

The patient had no operative or post-operative complications and tolerated post-op physical therapy well. He regained the majority of hip functionality by three months post-operatively and regained near-full mobility and functionality of the left hip by one year post-operative. Follow-up x-ray at one month post-operative showed the prosthesis to be in good position (Figure 4). Additionally, the patient’s LDL dropped from 190 to 73 after three months of statin therapy. The patient will continue to receive annual monitoring MRIs to evaluate the progression of AVN in the right hip.

## Discussion

AVN, also known as osteonecrosis, is a condition characterized by progressive subchondral bone death due to alteration or hypoperfusion of the relevant blood supply [1]. The etiology of this alteration in blood supply can be vast, but 75-90% of cases are due to long-term corticosteroid use, alcoholism, smoking, hip fractures and dislocations, and previous hip surgery. Less common causes include Caisson disease (the bends), human immunodeficiency virus (HIV), sickle cell disease, and Gaucher disease. However, hyperlipidemia is a known, yet uncommon, cause of the disease [2]. Furthermore, an LDL level greater than or equal to 190 mg/dL raises suspicion for a familial component or syndrome and meets current guideline criteria for high-intensity statin therapy for primary prevention of atherosclerotic cardiovascular disease [8]. The femoral heads are the most common location for AVN to occur due to the narrow caliber and fragile nature of the relevant arterial blood supply. Thus, it is a prime location for tiny cholesterol emboli to occlude.

Treatment of AVN of the hip is largely determined by the Ficat/Arlet staging system, which utilizes either plain film or MRI imaging to guide classification (see Table 1) [9,10]. Staging in AVN of the femoral head is an important step in the patient workup as it largely guides treatment decisions.

**Table 1:** Ficat/Arlet staging of avascular necrosis of the femoral head Ficat and Arlet staging [9,10].

Stage	Plain Film	MRI
0	Normal	Normal
I	Normal or mild osteopenia	Edema
II	Mixed osteopenia and sclerosis, subchondral cysts, no crescent sign	Significant marrow edema with associated geographic defect
III	Crescent sign, early signs of subchondral collapse	Same as plain film
IV	Subchondral collapse with secondary degenerative changes	Same as plain film

Due to the various possible etiologies of AVN, pharmacologic treatment has been largely unsuccessful in terms of revascularization [4]. There have been studies that looked at enoxaparin and other forms of anticoagulation in patients with AVN that have shown some promise but need further subgroup analyses [11]. Thus, treatment is often surgical in nature. The procedures can be split into THA and femoral head sparing procedures. Existing literature is conclusive that while THA can be considered in earlier stages, it is the definitive treatment once the femoral head has begun to collapse [4]. While THA has a high initial success rate, implantation in younger adults increases the probability of requiring revision surgery. One study found that in THAs in young adults with a mean age of 25.4, the mean time to revision was 10.1 years. The study also found that the main causes for revision were acetabular loosening, femoral loosening, and polyethylene wear [12,13]. Additionally, while some accommodations are occasionally made in the younger population, high-impact sports and activities that include running and jumping are usually discouraged as these can lead to faster wear of the prosthesis [14].

In patients with pre-collapse or stages I and II, a common treatment option is core decompression with cell/bone grafting. This comprises drilling several 8-10mm long narrow holes into the femoral head and often times grafting autologous marrow from the patient’s pelvis [4,15]. If successful, this relieves post-obstructive pressure while simultaneously introducing growth factors in an attempt to revascularize the bone. Meta-analyses have shown an overall success rate after core decompression of femoral heads to be approximately 65% [16]. The upside to this option is preserving the native bone and anatomy, which allows for the potential of a complete physical recovery and return to regular activity.

There is little consensus on how to screen or treat lipid levels in young adults under the age of 40. Until 2022, the United States Preventive Services Task Force (USPSTF) recommended screening men aged 35 and older for lipid disorders (A rating) and women aged 45 and older for lipid disorders who are also at increased risk for coronary artery disease (A rating) [17]. However, the 2018 multi-society guidelines for the management of blood cholesterol discuss the importance of screening for lipid abnormalities as a measure of assessing cardiovascular disease risk, including children, adolescents, and young adults. Specifically, the 2018 guidelines include a class IB recommendation for screening adults over the age of 20 with a lipid profile [8]. The limitation of the USPSTF recommendations is in the rare case of a young adult under 35 years old, like ours, who has no known medical history but has undiagnosed severe hyperlipidemia. Thus, this case highlights the need to screen for lipid disorders in patients of all ages, especially when their family history raises suspicion for cardiovascular disease or cardiovascular disease risk factors. Future research should consider the positives and negatives of asymptomatic lipid screening in young adults.

## Conclusions

AVN of the femoral head is an uncommon condition that is especially rare in the young adult population. If untreated, it can require invasive surgical intervention, usually THA. If caught prior to subchondral collapse, more conservative surgical measures can be attempted such as core decompression, to try and preserve the femoral head. Despite common risk factors being corticosteroid use, alcohol use, decompression sickness, and even idiopathy, this case points out the importance of considering previously undiagnosed hyperlipidemia as an etiology.
